# AraceaeDB: a functional genomics database of the Araceae family with a focus on konjac glucomannan biosynthesis in *Amorphophallus konjac* corms

**DOI:** 10.1093/hr/uhaf188

**Published:** 2025-07-17

**Authors:** Sen Chen, Yan Huang, DengGuo Tang, ZhiJian Long, Lucas Gutiérrez Rodríguez, LingMin Tian, Min Zeng, BoYa Wang, Xin Zhao, ShangLian Hu, Ying Cao

**Affiliations:** College of Life Science and Agri-forestrys, Southwest University of Science and Technology, No. 59, Middle Section of Qinglong Avenue, Fucheng District, Mianyang 621010, China; College of Life Science and Agri-forestrys, Southwest University of Science and Technology, No. 59, Middle Section of Qinglong Avenue, Fucheng District, Mianyang 621010, China; Engineering Research Center of Biomass Materials, Ministry of Education, College of Life Science and Agri-forestrys, Southwest University of Science and Technology, No. 59, Middle Section of Qinglong Avenue, Fucheng District, Mianyang 621010, China; College of Life Science and Agri-forestrys, Southwest University of Science and Technology, No. 59, Middle Section of Qinglong Avenue, Fucheng District, Mianyang 621010, China; College of Life Science and Agri-forestrys, Southwest University of Science and Technology, No. 59, Middle Section of Qinglong Avenue, Fucheng District, Mianyang 621010, China; Engineering Research Center of Biomass Materials, Ministry of Education, College of Life Science and Agri-forestrys, Southwest University of Science and Technology, No. 59, Middle Section of Qinglong Avenue, Fucheng District, Mianyang 621010, China; College of Life Science and Agri-forestrys, Southwest University of Science and Technology, No. 59, Middle Section of Qinglong Avenue, Fucheng District, Mianyang 621010, China; College of Life Science and Agri-forestrys, Southwest University of Science and Technology, No. 59, Middle Section of Qinglong Avenue, Fucheng District, Mianyang 621010, China; College of Life Science and Agri-forestrys, Southwest University of Science and Technology, No. 59, Middle Section of Qinglong Avenue, Fucheng District, Mianyang 621010, China; College of Life Science and Agri-forestrys, Southwest University of Science and Technology, No. 59, Middle Section of Qinglong Avenue, Fucheng District, Mianyang 621010, China; College of Life Science and Agri-forestrys, Southwest University of Science and Technology, No. 59, Middle Section of Qinglong Avenue, Fucheng District, Mianyang 621010, China; College of Life Science and Agri-forestrys, Southwest University of Science and Technology, No. 59, Middle Section of Qinglong Avenue, Fucheng District, Mianyang 621010, China

## Abstract

*Amorphophallus konjac*, as a significant representative of the Araceae family, demonstrates considerable potential for applications in medicine, healthcare, food, industry, and bioenergy due to its rich content of konjac glucomannan (KGM). However, the synthetic pathway of KGM remains largely unclear. Although genomic sequencing has been completed for various representative Araceae plants, including *Amorphophallus konjac*, a comprehensive data platform for deep analysis and exploration of the functions of these genes is lacking. In the current work, genomic and transcriptomic data from multiple Araceae species were integrated, and a database, AraceaeDB (http://www.araceaedb.com/), was constructed specifically for analyzing and comparing gene functions in Araceae plants. The gene functions in the database were annotated in detail, and their ortholog groups were identified and classified into different functional modules based on their expression patterns across various transcriptomic datasets. Multiple functional genomics analysis tools were developed, including OrthoGroup analysis, BLAST search, co-expression analysis, KEGG/GO enrichment analysis, and the JBrowse visualization tool. Moreover, the database incorporates several medicinally significant bioactive compounds traditionally important in the Araceae family, providing target prediction capabilities for these compounds. Furthermore, the major biosynthetic pathway of KGM has been successfully elucidated through these database resources, and a key gene *AkCSL3* has been identified. It has been further confirmed that overexpression of *AkCSL3* can significantly increase the content of KGM, suggesting its potential crucial role in the polymerization process of glucomannan in konjac corms.

## Introduction

In recent years, high-throughput sequencing technology has undergone rapid advancements, enabling a wide range of species to be subjected to high-precision genomic sequencing. This technological innovation has led to an explosion of large-scale, multi-dimensional omics data, including transcriptome, metabolome, proteome, and epigenome data, which harbor vast amounts of information reflecting the regulatory processes of growth and development, genetic variations, and molecular response mechanisms to environmental stresses in plants, animals, and humans. The surge in omics data has prompted the establishment of numerous integrated omics data platforms, such as gene expression profile database with knockdown/knockout of transcription (co-)factors in multiple species (KnockTF 2.0) [1], scATAC-seq database with known cell labels in multiple species (scATAC-Ref) [2], Solanaceae Information Resource (SoIR, https://soir.bio2db.com) [3], plant genomic information resources data-sharing platform (plantGIR, http://plantgir.cn/) [4], Plant Flowering-time Gene Database (PFGD, http://pfgd.bio2db.com/) [5], Plant Hormone Gene Database (PHGD) [6], Rice Genome Annotation Project Database (RGAP) [7], *Brassicaceae* Database (BRAD) [8], and LjaFGD database for the medicinal plant *Lonicera japonica* Thunb [9]. The construction of these integrated omics data platforms can facilitate standardized data management for sharing, as well as data mining and comparative analysis, thereby promoting the precise and efficient development of crop breeding technologies.

The family Araceae, comprising ~3645 species across 144 genera, stands as the third largest group within the monocotyledonous order Alismatales [10–12]. It exhibits rich morphological diversity and ecological adaptability, occupying important ecological niches in nature while also harboring significant economic value and research potential. For instance, *Arisaema heterophyllum* and *Pinellia ternata* are traditional Chinese medicines [13]; crops like *Amorphophallus konjac* (*A. konjac*) and *Colocasia esculenta* (*C. esculenta*) are excellent sources of glucomannan and starch [14, 15]; additionally, species, such as *Lemna minor* (*L. minor*) and *Spirodela polyrhiza* (*S. polyrhiza*), are important research subjects in environmental purification and bioenergy fields [16, 17]. Currently, the genomes and transcriptomes of representative Araceae species, including *A. konjac*, *L. minor*, *S. polyrhiza*, *C. esculenta, Pistia stratiotes* L, *Zantedeschia elliottiana*, and *Pinellia Tenore*, have been sequenced, providing valuable genetic information for in-depth studies of these plants [16, 18–23]. However, the lack of a unified platform for integrating this vast amount of data undoubtedly limits its further development and utilization, hindering breeding efforts for Araceae plants. Therefore, there is an urgent need to establish a comprehensive data integration platform in the future to fully explore the genetic potential of Araceae plants and promote the in-depth development of their breeding programs.

Konjac, an important crop in the Araceae family, is highly regarded for its corms, which are rich in konjac glucomannan (KGM). As a natural high-molecular-weight polysaccharide, KGM possesses unique physicochemical properties and physiological functions, making it widely applied in various fields such as food, medicine, and chemicals [24, 25]. To date, *Amorphophallus konjac* is the only species from which KGM can be commercially extracted in large scale. However, the synthetic pathway of KGM, particularly the process of how sucrose is partitioned to the corms and subsequently converted into KGM, remains unclear [18].

**Figure 1:**
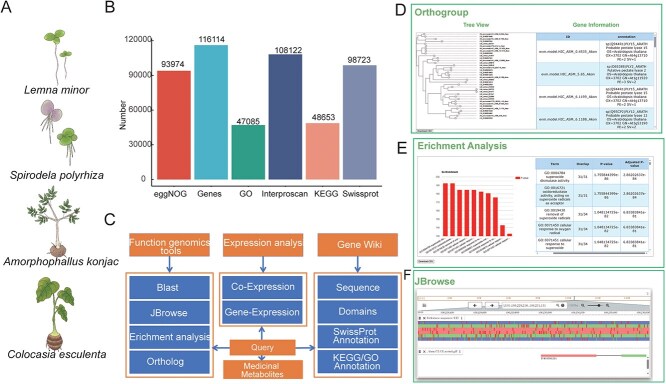
Overview of the Araceae functional genomics database. (A) Representative species of the Araceae family; (B) Annotation result of protein-coding genes; (C) The database structure; (D) A screenshot of the OrthoGroup query results, including the phylogenetic tree of the gene’s ontology system and the gene annotation information of the ontology members; (E) A screenshot of enrichment analysis results, presented with a visualized bar chart and a table; (F) A screenshot of the JBrowse query results.

In the current work, genomic data from seven species belonging to the Araceae family were collected, and four species, including *A. konjac*, *L. minor*, *S. polyrhiza*, and *C. esculenta*, were selected for subsequent analysis. The protein-coding genes of these species were annotated and classified based on homology using the Swiss-Prot, Kyoto Encyclopedia of Genes and Genomes (KEGG), and Gene Ontology (GO) databases. Additionally, extensive transcriptome data were gathered, and co-expression networks were constructed across multiple datasets utilizing Weighted Gene Co-expression Network Analysis (WGCNA). Furthermore, a suite of functional genomics analysis tools was developed and integrated into the AraceaeDB alongside the data. Given the medicinal significance of several Araceae species, the database also incorporates information on the active components of multiple traditional Chinese medicines (including *Pinellia ternata*, *Arisaema erubescens*, *Homalomena occulta*, *Acorus tatarinowii,* and *Typhonium rhizoma)*. Potential targets of these active components against the human proteome were predicted using deep learning models to facilitate the development of natural drugs. Finally, using *Amorphophallus konjac* as a model and leveraging the rich resources of AraceaeDB, an in-depth exploration of the biosynthetic pathway of KGM in konjac corms was conducted, aiming to elucidate the key metabolic pathways involved in carbon partitioning and conversion to KGM.

## Results

### Functions of AraceaeDB

Currently, gene function annotation and comprehensive analysis were performed on four nuclear genomes of *A. konjac*, *L. minor*, *S. polyrhiza*, *C. esculenta* from the AraceaeDB (Fig. 1A), as well as on 118 transcriptome sequencing (RNA-seq) datasets. As a highly intuitive and user-focused functional genomics analysis tool, AraceaeDB is structured around three core modules: [26] Functional Genomics Tools, [27] Co-expression Network, [28] Gene Wiki, and [24] Medicinal metabolites. These modules encompass a suite of interconnected sub-modules, seamlessly linked through sophisticated search utilities (Fig. 1C). The platform ensures that all analysis outcomes and query results are delivered to users in the form of clear images and comprehensive tables. These outputs are not only available for immediate download but also support various levels of interactive manipulation, enhancing the overall user experience and analytical capabilities.

#### Functional genomics tools

A comprehensive suite of functional genomics tools is offered by AraceaeDB, which is specifically designed to facilitate in-depth analysis and research of Araceae species. The BLAST tool incorporated within the platform enables homology searches to be conducted against protein-coding sequences derived from distinct species. The OrthoGroup function allows for the exploration of orthologs corresponding to specific genes, as well as their associated orthogroups, complete with evolutionary trees depicting these orthogroups (Fig. 1D). Furthermore, the tools are equipped to perform KEGG and GO enrichment analyses on gene sets of interest, empowering users to conduct such analyses (Fig. 1E). JBrowse is integrated to provide browsing capabilities for genomic and annotation information pertaining to these species (Fig. 1F).

**Figure 2:**
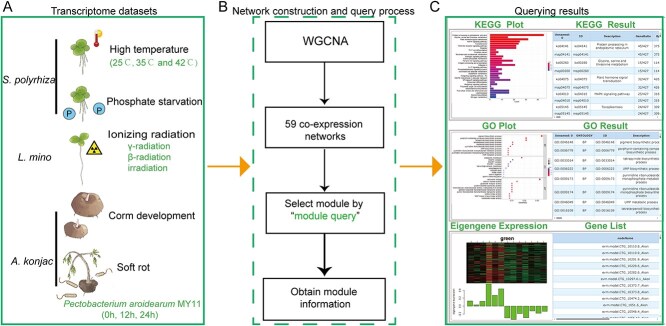
The construction of the co-expression network utilized transcriptome data from various Araceae species. (A) Schematic representation of the basic information of the dataset used for constructing the co-expression network. The transcriptome dataset primarily comprises data from *S. polyrhiza* exposed to various temperatures, *L. minor* exposed to different levels of ionizing radiation, different stages of corm development in *A. konjac*, and transcriptome data following infection by the konjac soft rot pathogen *Pectobacterium aroidearum* MY11. (B) Network construction methods and the query process of module information in the database. (C) Basic information of each co-expression module, including GO and KEGG annotation results of the genes contained in the module presented in pictures and tables, expression trend graphs of the module genes, and a list of the genes contained in the module genes.

A total of 116 114 protein-coding genes from *L. minor*, *S. polyrhiza*, *A. konjac*, and *C. esculenta* are annotated (Fig. 1A). Specifically, 98 723, 93 974, 108 122 of these genes have been annotated in the Swiss-Prot database, eggNOG-mapper database, and Interproscan database, respectively. Additionally, 47 085 genes have been classified under GO terms, while 48 653 genes are associated with pathway terms in the KEGG pathway terms (Fig. 1B). These genes are further categorized into 18 476 homolog groups, providing a rich data foundation for in-depth research. By integrating genome data, gene functional annotation, gene expression, and comparative genomic data, it provides robust support for functional genomics studies.

#### Co-expression network

Gene co-expression networks were constructed using multiple transcriptome datasets to investigate various biological processes (Fig. 2A). This integrated network encompasses studies on *S. polyrhiza* under normal temperature (25°C), moderate heat stress (35°C), and high heat stress conditions (42°C), as well as its response to phosphate deficiency [29, 30]. Additionally, it includes *L. minor* exposed to various forms of ionizing radiation, such as γ-radiation, β-radiation, and irradiation treatments [31]. Furthermore, the network covers different stages of corm development in *A. konjac*, from dormancy (Stage 1) through ‘changing head’ (Stage 2), corm expansion (Stage 3), to maturity (Stage 4) [18]. Also included within this unified framework is a specific analysis of *A. konjac’s* response to soft rot disease, comparing transcriptome samples from leaves pre-treated with or without *Bacillus velezensis* inoculation post-infection [32]. By analyzing these expression patterns, 59 co-expression modules were identified by WGCNA from these datasets. The ‘Model query’ function can be utilized by users to obtain detailed information about specific modules by inputting the dataset name and module name (Fig. 2B). This information includes the results of KEGG enrichment analysis, GO enrichment analysis, and expression pattern analysis for the genes within the selected module (Fig. 2C), allowing users to pinpoint the modules of interest to them.

#### Diversified search system

**Figure 3:**
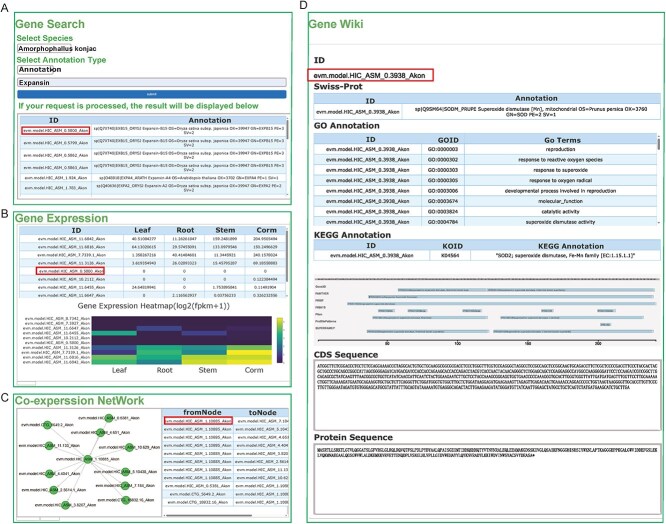
Screenshot of the query results and Gene Wiki page. (A) Taking the ‘*Expensin*’ gene as an example, the gene annotation query results include the genomic location, coding sequence (CDS), and related functional annotations; (B) Heatmap depicting the gene expression patterns of the *Expensin* gene across various samples or conditions, as identified by the gene ID obtained from the annotation query in (A); (C) Co-expression network analysis results for the *Expensin* gene, using the specific gene ID evm.model.HIC_ASM_0.5800_Akon. This network illustrates genes that exhibit similar expression profiles to Expensin, helping to elucidate potential functional interactions and pathways; (D) The results obtained from Gene Wiki include relevant information of *Expensin* gene, such as SwissProt annotations, GO/KEGG annotations, protein domains, CDS sequences, and protein sequences.

Various search methods are employed to facilitate access to gene function annotations, expression data, co-expression patterns, and orthogroups. The ‘Text Search’ and ‘Locus Search’ features allow users to enter keywords or Gene IDs (GIDs) to retrieve relevant gene information (Fig. 3A). In the ‘Gene Expression’ feature, users are able to conduct queries on the expression levels of genes within a specific species in a dataset, with results presented in tables and as log_2_-normalized FPKM heatmaps (Fig. 3B). The website’s ‘co-expression’ feature, allows users to input GIDs and explore their co-expression relationships across different networks. By setting a Weight Threshold Value, users can filter co-expression pairs and visualize them in an interactive manner on the page (Fig. 3C). Additionally, the ‘OrthoGroups’ feature, has been integrated into the Search System, enabling users to input GIDs and obtain relevant results.

#### Gene Wiki

The query results obtained from ‘Gene Expression’, ‘OrthoGroups’, ‘Co-Expression’, ‘Text Search’, and ‘Locus Search’ are linked to ‘Gene Wiki’ through GID. On these interfaces, users have the ability to click on the GID of genes, which directs them to the corresponding Gene Wiki page. This page comprehensively encompasses all annotation and sequence information pertaining to the gene within our database. It includes annotations from Swiss-Prot, KEGG, and GO, as well as domain information annotated by InterProScan, such as CDD, PANTHER, PRINTS, Pfam, ProSiteProfiles, and SUPERFAMILY (Fig. 3D). Furthermore, detailed information about a specific gene of interest can be obtained by users through the ‘GeneWiki’ interface.

#### Predicting targets of bioactives

**Figure 4:**
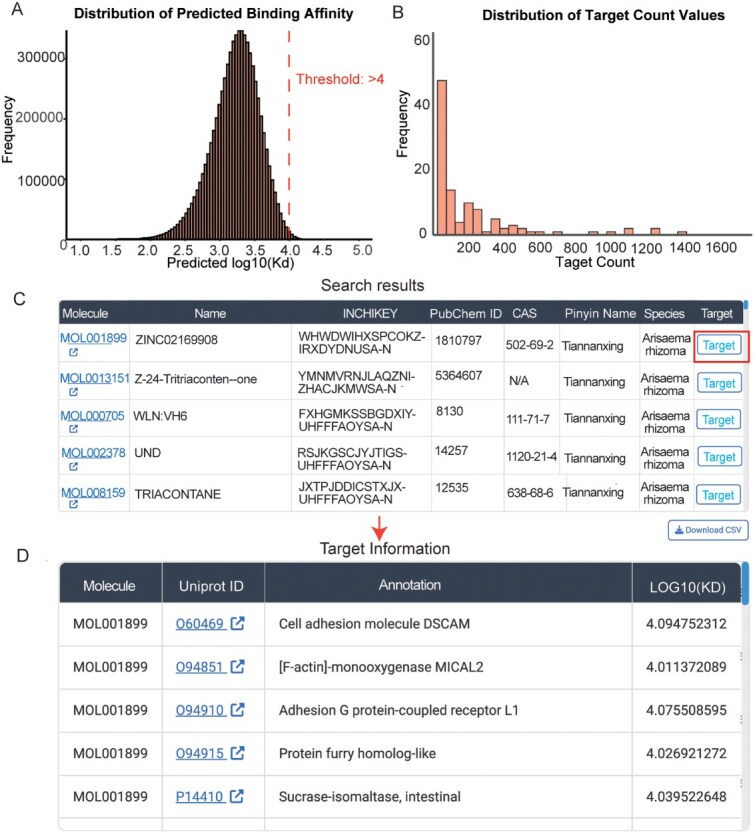
Prediction results of the affinity between Araceae metabolic small molecules and human protein-coding genes, along with screenshots of the query results in ‘Medicinal Metabolites’ module. (A) The distribution of predicted affinities (−log_10_(*Kd*)) between the 345 metabolic small molecules and 20 494 human proteins. Small molecule-protein pairs with −log_10_(*Kd*) ≥ 4 are considered to have potential interaction capabilities; (B) The number of potential target proteins for each small molecule; (C) Query results obtained through the ‘Medicinal Metabolites’ module using species names, enabling access to metabolite information for the queried species; (D) Target information for the metabolite MOL001899. Note: Users can obtain detailed information about a target metabolite by clicking on the ‘Molecule’ column in the search result interface to access molecular details. To retrieve target information for a molecule, users can click on the ‘Target’ column in the same interface. Additionally, by clicking on the ‘Uniprot’ column in the target information interface, users can access the complete functional annotation information of the corresponding protein in the UniProt database.

Using the deep learning-based small molecule-protein affinity prediction tool, deepDTA, the affinity between 345 deduplicated metabolic small molecules from five Araceae species and 20 494 human proteins was predicted. A total of 25 911 small molecule-protein pairs with potential binding capabilities (−log_10_(*Kd*) ≥ 4) were obtained (Fig. 4A). Among the prediction results, it was observed that most metabolic small molecules exhibited a potential binding target count ranging from 0 to 50, while only a small fraction of molecules were predicted to have over 1000 potential targets (Fig. 4B). Relevant small molecule information, including their target prediction details, can be queried through the ‘Medicinal Metabolites’ module of the database using species names (in Latin or Pinyin) or small molecule IDs (such as CAS numbers or InChIKey IDs) (Fig. 4C and D).

### A case study on konjac glucomannan biosynthesis in corms of *A. konjac*

#### Identification of key regulatory modules in co-expression network for KGM biosynthesis, starch biosynthesis, and corm expansion in *A. konjac*

**Figure 5:**
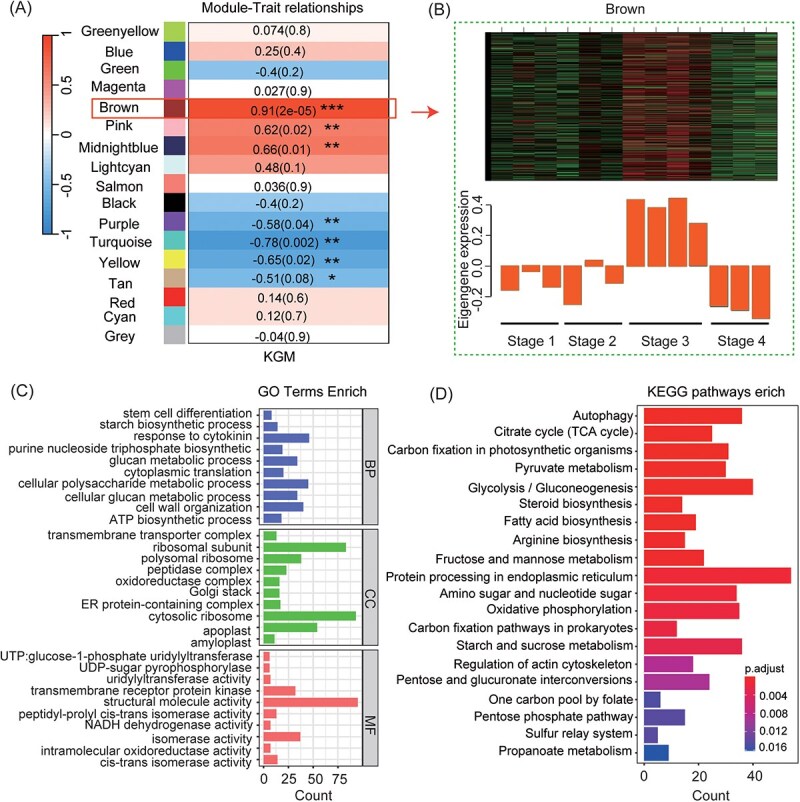
Identification process and analysis results of the KGM biosynthesis-related module. (A) heatmap showing the correlation between KGM content and modules through Pearson correlation analysis(^*^, *P* < 0.05; ^**^, *P* < 0.01, ^***^  *P* < 0.001); (B) expression pattern of these genes in Brown module; (C) GO enrichment results for the Brown module; (D) KEGG enrichment results for the Brown module. Note: The module highlighted with red border box represents significant enriched module associated with KGM content and selected for further analysis.

Correlation analysis was performed between the KGM content (obtained together with the transcriptome data of corm development) and the co-expression modules related to the expansion of *A. konjac* corms that resulted from WGCNA (Fig. 2). A module highly correlated with KGM content, named Brown (*r* = 0.91, *P* = 2e−05), was identified (Fig. 5A). Further analysis revealed that the gene expression levels within this module were significantly upregulated at the Stage 3, which corresponds to the corm expansion phase of *A. konjac,* compared to other stages, suggesting that the genes in this stage may be involved in the formation and accumulation of KGM in *A. konjac* (Fig. 5B). In this module, GO enrichment analysis revealed the biological process (BP) of these genes are mainly enriched in glucan metabolism, starch biosynthetic, cellular polysaccharide metabolism, and response to cytokinin. For the result of the cellular component (CC), it was revealed that these genes were mainly involved in ribosome. Moreover, in the molecular function (MF) analysis, these genes related to structural molecule activity, transmembrane receptor protein kinase, isomerase activity and UTP-monosaccharide-1-phosphate uridylyltransferase activity (Fig. 5C). Additionally, KEGG enrichment analysis indicated notable enrichment of genes involved in the Protein processing in endoplasmic reticulum, Glycolysis/Gluconeogenesis, Carbon fixation in photosynthetic organisms, Citrate cycle (TCA cycle), Fructose and mannose metabolism, Starch and sucrose metabolism in this module (Fig. 5D).

**Figure 6:**
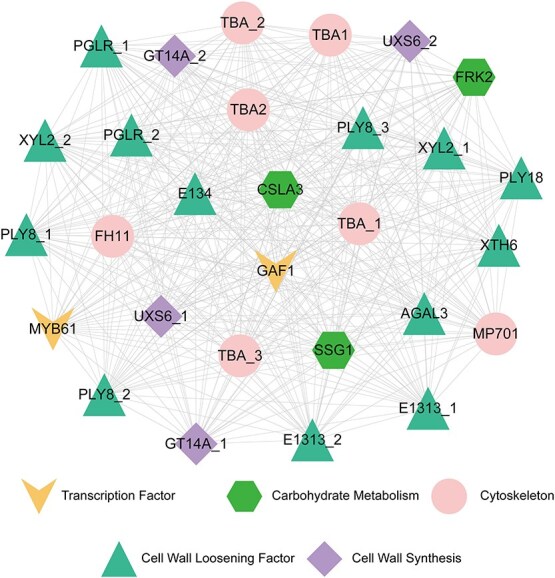
The co-expression network of transcription factors, as well as genes related to cell wall synthesis and sugar metabolism, among the 100 hubgenes.

In the Brown module, 100 core hub genes (hubgenes) were successfully identified and systematically classified based on their functional characteristics (Table S1, Fig. 6). Notably, only two transcription factors, *GAF1* and *MYB61*, were recognized, acting as positive regulators of the gibberellin signaling pathway [33], cell wall synthesis-related pathways[34], respectively (Fig. 6, Table S1). Furthermore, these two transcription factors were co-expressed with a series of cytoskeleton-related hubgenes, including *tubulin alpha chains* (*TBA*), *microtubule-associated protein 701* (*MP701*), and *formin-like protein 11* (*FH11*) [35], etc. Furthermore, they were found to be co-expressed with multiple hubgenes involved in cell loosening processes, such as *pectate lyase* (*PLY*), *β-xylosidase/α-l-arabinofuranosidase* (*XYL*), and *polygalacturonase* (*PGLR*), as well as others.

Additionally, three key hubgenes related to sugar metabolism were discovered. For instance, *fructokinase-2* (*FRK2*) serves as a crucial regulatory node in sugar metabolism. Another two genes encodes *glucomannan 4-β-mannosyltransferase 3* (*CSLA3*) and *granule-bound starch synthase 1* (*SSG1*), respectively, with the former being responsible for catalyzing the polymerization of glucomannan [36] and the latter catalyzing the synthesis of amylose [37]. These findings highlight the crucial role of this module in KGM and starch biosynthesis, as well as in the process of corm expansion in *A. konjac*.

#### Key gene involved in KGM and starch biosynthesis pathways in *A. konjac* corm

**Figure 7:**
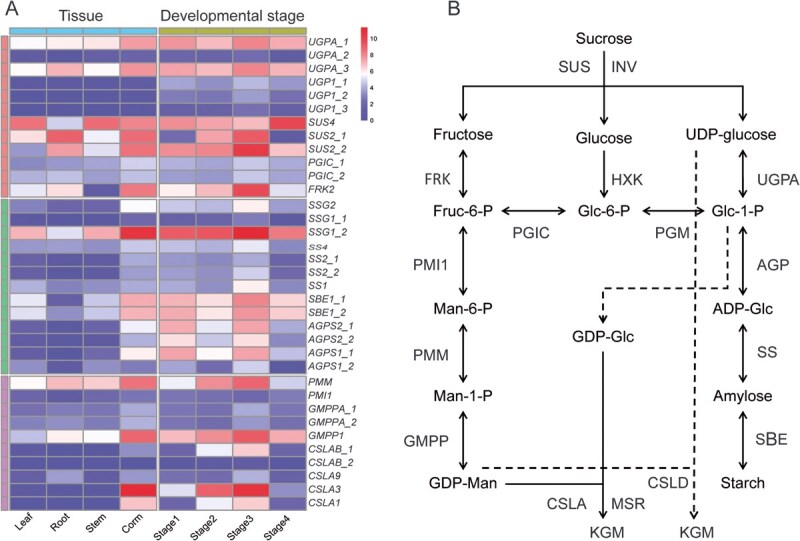
Heatmap and pathway diagram of gene expression involved in KGM and starch synthesis within the KGM biosynthesis-related pathway. (A) Heatmap of the expression of genes related to starch and glucomannan synthesis in different developmental stages and tissues of konjac corms; (B) the speculated synthesis pathway of KGM and starch in *A. konjac*.

In contrast to most plants that primarily store photosynthetic products as starch, *A. konjac* accumulates them predominantly as KGM in its corm. To identify key genes involved in corm KGM and starch biosynthesis, we examined the expression levels of genes within the Brown module. Six genes encoding enzymes that responsible for supplying substrates for KGM and starch biosynthesis, including *fructokinase 2* (*AkFRK2*), *UTP-glucose-1-phosphate uridylyltransferase* (*AkUGPA and AkUGP1*), *sucrose synthase* (*AkSUS 2 and 4*), and *Glucose-6-phosphate isomerase C* (*AkPGIC*) were identified, which exhibited specific or stronger expression in corms, particularly during the third stage of corm development (Fig. 7). Based on the same screening criteria, we identified eight genes specifically implicated in starch biosynthesis: *Glucose-1-phosphate adenylyltransferase large subunit 1 and 2 (AkAGPS1 and 2)*, *1,4-alpha-glucan-branching enzyme 1* (*AkSBE1*), *starch synthase* (*AkSS1, 2, and 4*), and *Granule-bound starch synthase* (*AkSSG1 and 2*). Additionally, eight genes specifically involved in KGM biosynthesis were also pinpointed, including *Phosphomannomutase* (*AkPMM*), *Mannose-6-phosphate isomerase 1* (*AkPMI1*), *Mannose-1-phosphate guanyltransferase alpha* (*AkGMPPA*), and *glucomannan 4-beta-mannosyltransferase* (*AkCSLA1, AkCSLA3,* and *AkCSLA9*) (Fig. 7). Interestingly, at the Stage 3, the expression levels of key genes involved in starch biosynthesis were lower than those involved in KGM biosynthesis. For instance, the FPKM value of the starch synthesis rate-limiting enzyme gene, *glucose-1-phosphate adenylyltransferase 11* (*AkAGPase11*), was only 172, whereas genes involved in KGM biosynthesis, such as *phosphomannomutase (AkPMM)* and *mannose-1-phosphate guanylyltransferase 1 (AKGMPP1)*, reached several hundred (450 and 586, respectively). Notably, the FPKM value of *AkCSLA3*, which is believed to encode glucomannan synthase [36], was as high as 1551. Although the *AkCSLA3* gene showed high expression levels during Stages 2 and 3, its expression dramatically decreased in Stage 4, with an FPKM value of only 9.3 (Table S3, Fig. 7A). These results indicate that *AKCSLA3* exhibits a unique expression pattern at different developmental stages of the konjac corm and may be involved in the synthesis of glucomannan during the Stage 3 (Fig. 7B, Table S2).

#### Characteristics and functional identification of *AkCSLA3*

**Figure 8:**
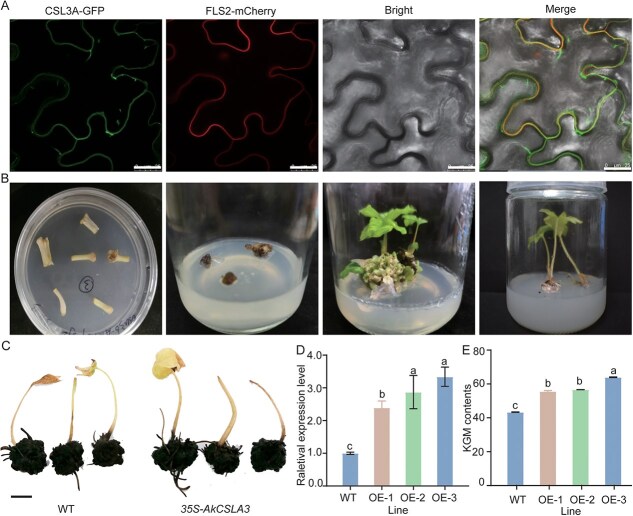
Overexpression of *AkCSLA3* promotes KGM accumulation of micro-corms in transgenic konjacs. (A) Subcellular localization of *AkCSLA3*, bars = 25 μm; (B) Generation of transgenic *A. konjac* plants overexpressing *AkCSLA3*; (C) Micro-corms of WT and *AkCSLA3* transgenic plants, bars = 1 cm; (D) qRT-PCR analyses of *35S*: *AkCSLA3* transgenic plants; (E) KGM content of micro-corms in WT and transgenic plants. Note: GFP fluorescent images were examined with a confocal microscope at 48 h after incubated in darkness; the results are presented as the mean values ± SE. Statistical significance was assessed by one-way ANOVA followed by Fisher’s LSD post hoc test (*P* < 0.05); OE represented overexpression of *AkCSLA3*.

To understand the genetic characteristics and functional role of *AkCSLA3*, a AkCSLA3: GPF fusion protein driven by the CaMV 35S promoter was constructed and transformed into tobacco epidermal cells to investigate the subcellular localization of AkCSLA3 in vivo. As shown in Fig. 8A, there is a significant overlap between the fluorescence signals of CSLA3-GFP and the membrane- located control protein (FLS2-mCherry), suggesting the membrane localization of AkCSLA3. Furthermore, AkCSLA3 was integrated into a plant expression vector, and then introduced into *A. konjac* by *Agrobacterium-*mediated transformation, yielding three transgenic plants with elevated expression levels (OE-1, OE-2, and OE-3) (Fig. 8B and C). A quantitative real-time PCR (qRT-PCR) analysis showed that the expression levels of *AkCSLA3* in these lines were 1.8-fold, 2.84-fold, and 3.3-fold higher than that in the wild type (WT) (*P* < 0.05), respectively (Fig. 8D). Furthermore, KGM contents in micro-corms were measured, and the results indicated that the KGM content in three overexpression lines increased by 28.27%–47.59% compared to the WT (Fig. 8E), demonstrating that overexpression of *AkCSLA3* gene can significantly enhance the KGM content in *A. konjac* corms.

## Discussion

### AraceaeDB: a feature-rich analysis platform for Araceae

AraceaeDB is a comprehensive functional genomics database designed to cater to the research needs of the scientific community. Currently, it contains genomic data from seven key species, as well as transcriptome data derived from 118 samples. These genetic resources are available for bulk download, filling a crucial gap in integrated platforms dedicated to the *Araceae* family. The database offers a suite of analytical tools that facilitate in-depth investigations into gene functional characteristics, expression patterns, and evolutionary traits. Currently, the AraceaeDB has only included genomic and transcriptome data for some plants within the Araceae family. However, the immense research and application value of the Araceae family dictates that more omics data will be generated in the future. AraceaeDB will continue to be updated, including the incorporation of genetic resources from more species and the optimization of user experience.

WGCNA clusters genes exhibiting similar expression profiles into co-expression modules. This approach is widely recognized for its ability to identify groups of co-expressed genes that potentially share biological functions [38]. Researchers leverage WGCNA to explore correlations between gene networks and phenotypes, identify hub genes within networks, and investigate developmental regulation in organs or tissues, as well as responses to biotic or abiotic stresses. Using various datasets, distinct co-expression networks have been constructed within AraceaeDB, revealing multiple modules containing regulatory insights into diverse biological pathways. Researchers can explore specific co-expression modules relevant to their research interests, integrating these findings with other database resources, tools, or analytical methods for comprehensive data analysis.

In recent years, advancements in virtual screening techniques and artificial intelligence (AI) technologies have significantly accelerated the process of drug development and new drug discovery[39, 40]. Multiple species within the Araceae family serve as traditional Chinese medicinal materials, holding immense development potential. Consequently, a functional module named ‘Medicinal Metabolites’ has been incorporated into our database. Through AI technology, potential small molecule-protein pairs have been screened, with the aim of facilitating the elucidation of the pharmacological properties of these Araceae species and supporting related drug development efforts.

### Biosynthesis process of KGM in *A. konjac* corms expansion stage

The biosynthesis of KGM and starch becomes closely linked with the corm’s enlargement. During the third and fourth stages of corm development, a large number of genes related to fructose, mannose, sucrose, and starch metabolism are highly expressed, along with significant enrichment of genes associated with response to cytokinin and cell wall organization. Network analysis emphasizes *AkGAF1* as a central hub gene within the Brown module, co-expressed with numerous cell expansion factors (Fig. 6A). The role of the gibberellin (GA) signaling pathway in plant cell division and expansion has been extensively documented [41], and in *Arabidopsis*, *AtGAF1* has been shown to promote internode elongation [42]. Therefore, it is plausible to speculate that *AkGAF1* may regulate processes such as KGM and starch biosynthesis, as well as corm enlargement through the GA signaling pathway.

In the corms of *konjac*, the exact pathway through which sucrose is converted into KGM remains incompletely understood. However, existing research suggests three possible routes for this conversion after sucrose is transported from photosynthetic tissues to the corms [15, 43, 44]. Generally, sucrose is hydrolyzed into fructose and glucose, which undergo a series of enzymatic reactions to form GDP-mannose and GDP-glucose, ultimately being polymerized into KGM. Among the three possible pathways, several key differences remain unclear. Firstly, it is uncertain whether sucrose hydrolysis is catalyzed by invertase (INV) or sucrose synthase (SUS). Additionally, the substrate for GDP-mannose formation, whether fructose or glucose, remains undetermined. Finally, the precise enzyme catalyzing the polymerization of GDP-mannose and GDP-glucose, whether CSLA, Cellulose synthase-like protein D (CSLD), MANNAN SYNTHESIS-RELATED (MSR) or another, is still unknown.

In this study, it was observed that *AkSus2, AkSus4, AKUGPA, AKUGP1,* and *FRK2* were highly expressed during the corm expansion stage, and all these genes were encompassed within the Brown module (Fig. 7A). In contrast, *AkINV* and *AkHXK* were not found within the Brown module, while all *AkINA* genes consistently exhibited low expression throughout corm development (Table S3). These findings suggested that sucrose conversion in *A. konjac* corms was predominantly catalyzed by SUS, providing substrates for the synthesis of KGM and starch (Fig. 7B). This conclusion was supported by other studies, which demonstrated that during potato tuber formation, there was a reduction in cell wall INV activity and an increase in SUS activity [26, 45, 46]. Additionally, high expression levels of *CSLD* and *MSR1* was not detected during the corm expansion stage. Instead, it was found that multiple *AkCSLAs* genes, particularly *AkCSLA3*, were upregulated (Fig. 7A), indicating the potential major role in the final step of KGM polymerization in *A. konjac* corms.

### *AkCSLA3*, a key gene in the biosynthesis of KGM in *A. konjac* corms

There is significant variation in the content of KGM among different varieties of *konjac* corms. In *A. konjac*, mature corms contain as much as 55% to 60% KGM in dry matter [47], whereas in other varieties, such as *A. paeoniifolius*, the KGM content is lower, with starch being the predominant component in their corms [48]. This suggests that there may be mechanisms in *A. konjac* that direct more sucrose, transported from aboveground parts, towards the biosynthesis of KGM.

In the current work, KGM and starch biosynthesis in *A. konjac* corms appear to be synchronous, predominantly occurring during the corm expansion stage. Key genes involved in KGM biosynthesis, especially *AkCSLA3*, show significantly higher expression during Stages 3 compared to rate-limiting enzyme gene *AGPases*. This differential expression pattern likely contributes to the high accumulation of KGM in *A. konjac* corms. Notably, *AkCSLA3* expression is minimal and is lower than that of genes involved in starch biosynthesis in Stage 4 corms (Fig. 7A), which may explain the observed decrease in KGM content during Stage 4. These findings suggest that *AkCSLA3* plays a crucial role in the high accumulation of KGM in konjac corms, not only as a key enzyme in the final step of KGM polymerization but also by controlling the redirection of sucrose from aboveground photosynthates towards KGM biosynthesis during the corm expansion stage. *AkCSLA3* maintains high expression levels throughout corm development, and through genetic transformation, its expression levels in dormant corms were successfully increased by 1.8–3.4 times, resulting in a 20%–27% increase in KGM content in corms (Fig. 8). This finding highlights the crucial role of the *AkCSLA3* gene in promoting KGM accumulation.

Although the Golgi apparatus is the central site for the synthesis of most plant hemicelluloses, this study found that AkCSLA3 may be localized at the plasma membrane (Fig. 8A). This finding is consistent with previous research in yeast, where *A. konjac* AkCSLA3 was localized at the plasma membrane, enabling the yeast to acquire the ability to synthesize KGM and deposit it in the extracellular matrix. The subcellular distribution characteristics of AkCSLA3 likely result in tightly adhered patches of KGM on KGM idioblast cell membranes [49], whereas CSLA localized to the Golgi apparatus may primarily participate in cellulose KGM biosynthesis.

Additionally, it is noteworthy that BsCSLAs from *Bletilla striata*, which are localized in the Golgi apparatus, participate in the synthesis of *Bletilla* polysaccharides (which are water-soluble polysaccharides deposited only in the pseudobulbs, with glucomannan as the main component) [50]. These polysaccharides are distributed within the cytoplasm inside the cell membrane rather than in the cell wall. Different subcellular localizations influence distinct enzymatic reactions. These results suggesting that the functional differentiation of the cellulose synthase-like family A (CSLA) may be a key factor in the flow of glucomannan towards highly polymerized glucomannan, water-soluble polysaccharides, or cell wall components.

In subsequent research, the *AkCSL3* gene will be further precisely modified utilizing gene editing technologies such as CRISPR-Cas9, with the aim of enhancing the content of KGM or improving its quality attributes, including molecular weight distribution and viscosity characteristics. Simultaneously, the binding modes, catalytic efficiency, and substrate selectivity (UDP-Glc/UDP-Man ratio) of AkCSL3 with its substrates (UDP-Glc and UDP-Man) will be evaluated through three-dimensional structural modeling, molecular docking, site-directed mutagenesis, and in vitro enzyme activity assays. Although our ultimate goal is to promote the industrial application of transgenic konjac, throughout this process, we will continuously monitor and assess the potential ecological effects of transgenic konjac in natural environments, such as the impact of gene flow on wild related species and the potential influence on the structure and function of soil microbial communities, ensuring the comprehensiveness and responsibility of our research.

## Conclusions

In summary, the genomes of representative species from the Araceae family, along with extensive transcriptome data, were collected in this study, leading to the development of a functional genomic database for the Araceae family (AraceaeDB). Furthermore, to facilitate the development and utilization of natural drugs, a drug target prediction tool was integrated into the database. It is hoped that this addition will contribute to the pharmacological analysis of Araceae-derived drugs and the development of active substances. Subsequently, the resources within AraceaeDB were utilized to conduct an in-depth exploration of the biosynthetic pathway of KGM in konjac corms. Based on the results of this study, it is hypothesized that in konjac corms, sucrose is decomposed into glucose and fructose by SUS rather than IVA. Finally, GDP-mannose and GDP-glucose are polymerized into KGM by AkCSLA, with AkCSLA3, in particular, potentially playing a significant role in this process. The results shown in this study suggests that a great potential of *AkCSLA3* in the genetic improvement of KGM biosynthesis and quality in konjac.

## Materials and methods

### The establishment and functions of the AraceaeDB database

#### Data source

The genomic data for both *L. minor* (identifier ID 27408) and *S. polyrhiza* (with identifier ID 55812) were retrieved from the CoGE website. Genomic data for *A. konjac* were downloaded from NCBI, carrying the accession number GCA_022559845.1. Similarly, genomic data for *C. esculenta, P. pedatisecta* and *Pistia stratiotes* L were obtained from the China National GeneBank DataBase (CNGD) with the accession number CNP0001082, CNP0003127 and CNP0002271. *Zantedeschia elliottiana* annotation files were download from Figshare (https://doi.org/10.6084/m9.figshare.22656112) (Table S4). Transcriptomic data, excluding unpublished datasets related to various tissues and infection with soft rot in *A. konjac*, were retrieved from the National Center for Biotechnology Information (NCBI), Bioproject Database, GEO Database, and SRA Database (Table S5). The drug small molecule information of five species of the Araceae family (*Pinellia ternata*, *Arisaema erubescens*, *Homalomena occulta*, *Acorus tatarinowii, and Typhonium rhizoma*) was sourced from the TCMSP (Whole Genome) database [51]. The human whole-genome protein data was obtained from the UniProt website.

#### Gene function annotation

Protein-coding genes were extracted from four species [52] using the Tbtools software. A BLAST database was created using Swiss-Prot [53], and BLASTP alignments were performed to annotate the protein-coding genes with Swiss-Prot annotations. The tool described by Cantalapiedra *et al*. [54] was utilized to acquire GO [55] and KEGG [56] annotations for the genes. Additionally, protein domains were annotated using the InterProScan [57] online software. Finally, the protein-coding genes of these species were classified into different ortholog groups using OrthoFinder software [58].

### Transcriptome analysis and co-expression network construction

Firstly, FastQC software was used for quality control of RNA-seq data [28]. Subsequently, RNA-seq reads were mapped to the genome using Bowtie2 [59], and gene expression quantification in FPKM (Fragments Per Kb of exon per Million mapped fragments) was computed using StringTie [60]. Co-expression network was constructed using the R package WGCNA [61], and network visualization was performed using Cytoscape software [62]. For each co-expression module, gene enrichment analysis for KEGG pathways and GO terms was conducted using the AnnotationForge and ClusterProfiler R packages [63].

#### Database construction

All pre-processed data, encompassing genetic resources such as gene functional annotation information, gene co-expression pairs, gene co-expression module information, gene expression datasets, and sequence information, were integrated into a MySQL database. The Flask framework was utilized to facilitate data querying, processing, and transfer between the website and the database.

### Website interaction and tool construction

The jQuery framework and Ajax technology were utilized to facilitate interactive data exchange between our website’s views and data. Transcriptome heatmaps were visualized using the Plotly.js library. For the visualization of genome features, JBrowse2-web [64] was employed. The viroblast web server [65] was used to implement our BLAST service. Evolutionary relationships within each orthogroup were visualized using phyloview.js. Gene co-expression networks were visualized with cytoscape.js [66]. The Python package gseapy [67] was utilized for GO and KEGG enrichment analysis, and the enrichment results were visualized on web pages using the Google Charts JavaScript library.

### Prediction of affinity between small molecules and proteins

The molecule structure files obtained from TCMSP were converted from the PDB (Protein Data Bank) format to the SMILES (Simplified Molecular Input Line Entry System) format using Open Babel [68]. Subsequently, the pre - trained model based on the PDBBind dataset (available at https://github.com/KSUN63/DeepDTA-Pytorch) [69] was loaded using the deepDTA tool [70] to predict the −log_10_(*Kd*) values of the interactions between the small molecules and human protein targets derived from the UniProt reference proteome (UP000005640).

### Characteristics and functional identification of *AkCSLA3*

#### **Plant material and growth condition**s

*A. konjac* used for plant transformation in this research were collected from the Botanical Resource Center of Southwest University of Science and Technology, Mianyang, Sichuan, China. *A. konjac* plantlets were grown on fresh Murashige and Skoog medium (MS) at 25°C under a 16/8 h light/dark cycle with 2000 lux supplemental light at a relative humidity of ~60% for optimum growth. Each of the following analyses were performed on at least three separate genotypes of each transgenic line and control plants.

### Transgenic vector construction and plant transformation

The *AkCSLA3* gene were clone from corm of *A. konjac*. Then *AkCSLA3* was inserted into the modified vectors pCAMBIA1303 and pCAMBIA1300-35S-GFP to generate the constructs pCAMBIA1303-35S*:AkCSLA3* (35S:*AkCSLA3*) and pCAMBIA1300-35S:*AkCSLA3-GFP* (35S:*CSLA3-GFP*), respectively, and then introduced into *Agrobacterium tumefaciens* strain EHA105 for transformation.

The leaf petioles of wild-type *A. konjac* sterile seedling were cut into segments ~1 cm in length and subsequently placed into induction medium that consisted of MS medium supplemented with 30 g/l sucrose, 1 mg/l 6-benzylaminopurine (6-BA), and 0.1 mg/l 1-naphthaleneacetic acid (NAA), with the pH adjusted to 5.6 to 5.8. The segments were cultured for ~40 days until callus induction, and subsequently transformed using *Agrobacterium tumefaciens* strain EHA105. After 48 hours of dark culture on co-cultivation MS medium, 30 g/l sucrose, and 10 mg/l acetosyringone (AS), the explants were transferred to selection medium (MS medium, 30 g/l sucrose, 1 mg/l 6-BA, 0.1 mg/l NAA, 40 mg/l Carb, 9 mg/l Hyg, pH 5.6–5.8) until plantlet regeneration. Finally, the regenerated plantlets were transferred to corm induction medium (1/2MS medium, 30 g/l sucrose, 0.08 mg/l NAA, pH 5.6–5.8) to induce the formation of micro-corms in tubes.

### Extraction of plant Total RNA, reverse transcription, and qRT-PCR

Using the RNAprep Pure Plant Plus Kit (Polysaccharides and Polyphenolics-rich) (Tiangen Biotech), total RNA was extracted from corm, stem, root, and leaf tissues of *A. konjac*. The extracted RNA was reverse transcribed into cDNA using the HiScript III 1st Strand cDNA Synthesis Kit (+gDNA wiper) (Vazyme Biotech).The qRT-PCR was performed using the Taq Pro Universal SYBR qPCR Master Mix (Vazyme Biotech) and run on a CFX connect™ Real-Time PCR detection system (Bio-Rad, Singapore). The konjac *eukaryotic translation initiation factor 4A (EIF4A*) gene was used as an internal standard and the gene-specific primers are listed in Supplemental data (Table S6).

### Subcellular localization

The fusion construct (35S:CSLA3-GFP) and a membrane location marker vector (35S:FLS2-mCherry) were separately introduced into *Agrobacterium* GV3101. *Agrobacterium*-mediated transient expression in *Nicotiana benthamiana* leaves was performed as described. The *Agrobacterium* cells carrying a given construct were collected and re-suspended in MES buffer, and then infiltrated into 1-month-old tobacco leaves using a 10 ml syringe. Following this, the infiltrated leaves were incubated in darkness for 48 h before being observed under a confocal laser scanning microscope.

### **Measurement of konjac glucomannan conten**t

After harvesting, the fresh konjac (*A. konjac*) corms were dried, weighed, and then ground into powder. An appropriate amount of sample was taken and added to a 0.1 mol/l formic acid-sodium hydroxide buffer. The mixture was stirred at 30°C for 4 hours, followed by centrifugation at 4000 rpm for 20 minutes to collect the supernatant containing KGM. The KGM extract was then subjected to acid hydrolysis by adding sulfuric acid and heating. After cooling, 6 mol/l sodium hydroxide was added to obtain the KGM hydrolysate. The KGM content was determined using the DNS method by measuring the absorbance at 550 nm. A glucose standard curve was constructed using the procedure as described. The formula for calculating the KGM content in the sample is as follows:


\begin{align*} KGM\left(\%\right)=\frac{\varepsilon \left(5T-T0\right)\times 50}{m\times \left(1-\omega \right)\times 1000}\times 100 \end{align*}

where the $\varepsilon$ represents the ratio of the molecular weights of the glucose and galactose residues in KGM to their molecular weights after hydrolysis, which equals 0.9. Here, *T* represents the milligrams (mg) of glucose in the hydrolysate of KGMs, *T0* represents the milligrams (mg) of glucose in the extract of KGM, denotes the mass of the sample taken in grams (g), and $\omega$ signifies the moisture content of the sample.

## Supplementary Material

Web_Material_uhaf188

## Data Availability

The supplementary material contains comprehensive data that substantiates the conclusions drawn from this study.
